# The global epidemiology of alcohol-associated liver disease

**DOI:** 10.1097/HC9.0000000000000947

**Published:** 2026-04-24

**Authors:** Pojsakorn Danpanichkul, Francisco Idalsoaga, Frank Murray, Juan Pablo Arab, Luis Antonio Díaz

**Affiliations:** 1 Department of Internal Medicine, Texas Tech University Health Sciences Center, Lubbock, Texas, USA; 2 Departamento de Gastroenterología, Escuela de Medicina, Pontificia Universidad Católica de Chile, Santiago, Chile; 3 Division of Gastroenterology and Hepatology, Western University and London Health Sciences Centre, London, Canada; 4 Department of Medicine, Royal College of Surgeons in Ireland, Beaumont Hospital, Dublin 9, Ireland; 5 Division of Gastroenterology, Hepatology, and Nutrition, Department of Internal Medicine, Virginia Commonwealth University School of Medicine, Richmond, Virginia, USA; 6 MASLD Research Center, Division of Gastroenterology and Hepatology, University of California San Diego, San Diego, California, USA

**Keywords:** alcohol-associated liver disease, alcoholic cirrhosis, epidemiology, fatty liver, health policy, public health policies

## Abstract

Alcohol-associated harm remains a major, largely preventable driver of global morbidity, mortality, and societal costs, and alcohol-associated liver disease (ALD) is a major consequence of long-term exposure. According to the World Health Organization, average consumption was 5.0 L of pure alcohol per person aged ≥15 years in 2022, with 21% unrecorded consumption and substantial regional variation. Per-capita intake declined from 5.7 L in 2010 to 5.0 L in 2022, yet current models forecast an increase to 5.7 L by 2030. In 2023, an estimated 3.15 million people were living with alcohol-associated cirrhosis, and 419,429 new cases occurred; while crude burden has risen, age-standardized prevalence was 34.6 per 100,000, reflecting demographic shifts. Age-standardized mortality due to ALD cirrhosis was highest in the European Region (5.8 per 100,000) and the Americas (5.0 per 100,000), and projections suggest crude ALD mortality will increase by 76% from 2021 to 2050 (354,200–624,150). Because alcohol exposure is frequently underreported, ascertainment using validated questionnaires and biomarkers is critical for surveillance and research. Scalable models linking alcohol-use detection with staged fibrosis assessment and triage pathways can narrow diagnostic gaps and support health system planning. Coupling these pathways with the implementation of high-impact “best-buy” alcohol control policies is essential to reduce the global ALD burden.

## Graphical abstract

**Figure:**
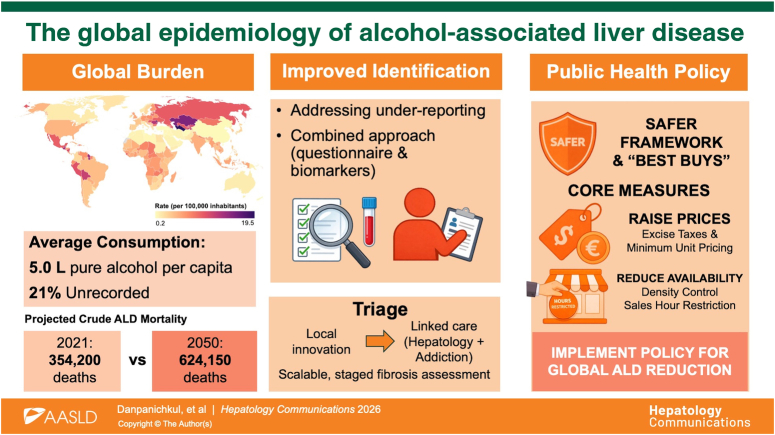

## INTRODUCTION

Alcohol-associated harm remains a major and largely preventable driver of global morbidity, mortality, and societal costs, extending well beyond health outcomes to include injuries, violence, and productivity losses.^1^^–^^3^ Within this broader burden, alcohol-associated liver disease (ALD) represents one of the clearest manifestations of long-term alcohol exposure and a major contributor to premature death and disability.^3^^,^^4^ Over the past 3 decades, the prevalence of ALD and alcohol-attributable primary liver cancer has continued to rise, underscoring that the global liver health burden is worsening despite the availability of effective prevention strategies.^3^ Alcohol exposure often interacts with other major risk factors for chronic liver disease, including type 2 diabetes mellitus (T2DM) and obesity.^5^ Moreover, alcohol can also cause new infections with hepatitis B and C, and promote an accelerated progression of metabolic dysfunction–associated steatotic liver disease (MASLD) to advanced fibrosis stages.^6^

In this review, we synthesize contemporary evidence on the global epidemiology of ALD, summarizing temporal trends and geographic variation in prevalence and mortality, and highlighting key methodological limitations that can bias burden estimates. Because alcohol exposure is often underreported in clinical and observational data, we discuss more robust approaches to exposure ascertainment (eg, validated questionnaires and objective biomarkers) and their implications for surveillance. We also outline scalable strategies—combining staged fibrosis assessment and triage pathways, to improve case finding, risk stratification, and health system planning. Finally, we discuss high-impact (“best-buy”) alcohol control policies that could benefit people at risk for and living with ALD.

## GLOBAL ALCOHOL INTAKE AND ITS ECONOMIC IMPACT

Overall, alcohol is responsible for about 5% of all global deaths and disability, making it one of the leading modifiable risk factors for disease burden worldwide.^1^ According to the World Health Organization (WHO), the global population consumed an average of 5.0 L of pure alcohol per person per year (age ≥15) in 2022.^7^ In the same period, the European WHO Region had the highest per-capita alcohol intake (9.1 L), followed by the Region of the Americas (8.0 L). In contrast, the Eastern Mediterranean, African, and Southeast Asia WHO Regions had the lowest per-capita alcohol intake (0.2, 3.5, and 3.6 L, respectively).^1^ About 21% of all alcohol consumed worldwide is unrecorded, including home-brewed alcohol, illicit production, and other sources.^1^ Globally, alcohol intake per capita has declined over the last decade (from 5.7 L in 2010 to 5.0 L in 2022), but it remains high and may rise in the absence of stronger interventions. Currently, the WHO forecasts that global per-adult consumption could reach up to 5.7 L by 2030, falling short of targets to reduce harmful use.^8^

Although 56% of adults report abstaining from alcohol in 2019,^1^ a substantial number of current drinkers do so at harmful levels. Among current drinkers, the average consumption is 27 g of pure alcohol per day, roughly equivalent to 2 standard drinks (United States/Canada) daily.^1^ Such patterns far exceed healthy limits and fuel alcohol use disorders (AUD), a harmful pattern of alcohol use that seriously impairs health and functioning, and related harms and deaths.^9^ WHO has estimated that 400 million people globally (about 7% of the adult population) were living with an AUD in 2019.^1^ According to Global Burden of Disease (GBD) estimates, the prevalence of AUD is highest in regions with heavy drinking patterns; for example, Eastern Europe has the greatest burden of AUD, with an age-standardized prevalence of over 3200 per 100,000 population.^3^ Globally, men are 3 times more likely to consume alcohol and develop AUD than women. This is reflected in the associated death rates in 2019, which were 2 million in men compared with 0.6 million in women.^10^ However, worrying trends of increased drinking, ALD, and AUD among women have been observed in some regions, narrowing the historical gender gap.^11^^,^^12^

**FIGURE 1:**
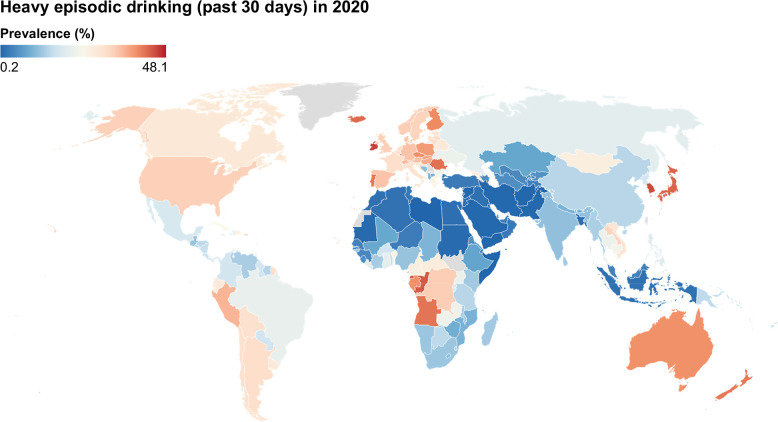
Age-standardized prevalence of heavy episodic drinking in individuals aged over 15 years in the past 30 days (2020). Data were obtained from the World Health Organization (WHO) Global Health Observatory.

Heavy episodic (binge) drinking is defined by an intake of ≥ 60 g of pure alcohol on at least one occasion in the past month, a common pattern that correlates with the development of alcohol harms.^13^ According to the WHO, about 38% of current drinkers report periodic heavy episodic (binge) drinking. This pattern of consumption is particularly prevalent among young adults and in Europe, where nearly 1 in 2 drinkers binge (Figure 1).^1^ Those aged 20–39 years experience a disproportionately high proportion of alcohol’s harms, accounting for about 13% of all alcohol-attributable deaths that occur in young adults.^1^ Earlier initiation and sustained alcohol intake contribute substantially to the later development of ALD, the prevalence of which rises sharply with age.^14^ A prior study using GBD data reported a 19-fold increase in ALD prevalence from 15–19 to 20–24 years, with a further 2.5-fold increase to 25–29 years.^15^ Children and adolescents are especially vulnerable to commercial influences and social pressures that promote alcohol use. Their heightened sensitivity to dopamine-related reward pathways may further reinforce continued consumption.^16^ Young adults experienced a faster increase in ALD-related hospitalizations and worsening disease severity than older adults, based on analyses of the National Inpatient Sample.^17^ While these findings highlight a concerning increase among young adults, the overall burden of ALD, including both prevalence and mortality, remains highest among older adults, underscoring their continued vulnerability and cumulative harmful effect of alcohol in the liver.^18^ Moreover, older adults with ALD show increasing trends of multiple psychiatric comorbidities, with larger rises than in younger patients, especially for major depressive disorder, anxiety, and bipolar disorder.^19^

## CURRENT GLOBAL AND REGIONAL BURDEN OF ALD AND PROJECTIONS TO 2050

### The natural history of ALD

Approximately 26% of individuals with hazardous drinking and 55% of those with AUD will develop some form of ALD.^20^ In a systematic review of 35 studies that included 513,278 individuals, the prevalence of ALD was estimated to be 3.5% in the general population.^21^ In addition, the 10-year incidence of ALD is estimated to be 0.6%.^22^ In individuals seeking treatment for AUD, a recent cohort study demonstrated a 7.1% cumulative incidence of ALD after 10 years, increasing to 13% in those with T2DM.^22^ About 10%–20% of individuals with ALD will develop cirrhosis. Thus, the prevalence of alcohol-associated cirrhosis is estimated at ~0.3% in the general population, but rises to 12.9% in those with AUD.^21^ In addition, the risk of hepatocellular carcinoma (HCC) among individuals with cirrhosis is estimated at 1%, 3%, and 9% at 1, 5, and 10 years.^23^ Given the high levels of alcohol use, prevalence of AUD, and the underreporting of alcohol consumption, it is likely that the prevalence of ALD is vastly underestimated in the overall population.

Compared with MASLD, individuals with ALD had a higher proportion of advanced fibrosis and cirrhosis.^24^ ALD may progress from steatosis to steatohepatitis, fibrosis, and cirrhosis, with decompensation, liver failure, and death.^25^ In alcohol-associated cirrhosis, HCC occurs at a rate of 0.9%–5.6% per year, and long-term heavy alcohol use markedly increases risk.^26^ ALD is also linked to substantially higher mortality from infection, cardiovascular disease, and non-hepatic cancers, as well as metabolic, neurologic, and reproductive comorbidities.^27^^,^^28^

### Global and regional prevalence of alcohol-associated cirrhosis

**FIGURE 2:**
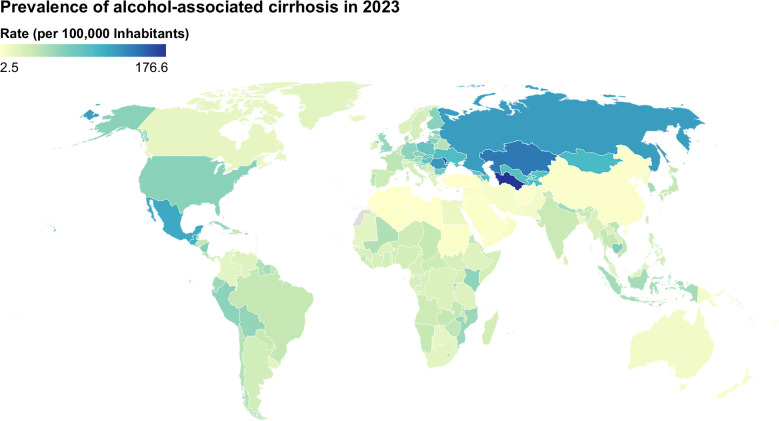
Age-standardized prevalence of alcohol-associated cirrhosis (2023). Data were obtained from the Global Burden of Disease database (https://vizhub.healthdata.org/gbd-results/).

In 2023, an estimated 3.15 million people were living with alcohol-associated cirrhosis globally.^29^^,^^30^ About 419,429 new cases of alcohol-associated cirrhosis worldwide developed that year, which is 18.3% higher than in 2000. However, the age-standardized prevalence has been estimated at 34.6 per 100,000 inhabitants in 2023, representing a 29.0% drop since 1990 (Figure 2). Thus, these differences between crude and age-standardized estimations reflect substantial demographic changes over the last 3 decades. There are important differences in age-standardized prevalence by sex in 2023, with rates estimated at 47.0 and 22.7 per 100,000 inhabitants in men and women, respectively. Between 1990 and 2023, men and women exhibited decreases of 27% and 32% in age-standardized prevalence, respectively.^29^^,^^30^

According to GBD estimates, the European WHO Region has the highest prevalence of alcohol-associated cirrhosis, at 67.5 per 100,000 in 2023, followed by the Region of the Americas at 58.4 per 100,000 inhabitants (Figure 2).^29^^,^^30^ This is consistent with the very high alcohol consumption in Europe (particularly Eastern Europe) and the Americas.^31^ In contrast, the lowest age-standardized prevalence is observed in the Eastern Mediterranean and Western Pacific WHO Regions, with 8.8 and 16.9 per 100,000 inhabitants, respectively. According to the sociodemographic Index (SDI)—a composite of income, education, and fertility rates^32^^,^^33^—the highest age-standardized prevalence was observed in countries with high SDI (40.4 per 100,000 inhabitants), while the lowest was observed in high-middle and low SDI countries (29.4 and 29.5 per 100,000 inhabitants, respectively). The contribution of low-, lower-middle-, and middle-SDI countries to the global burden of ALD has significantly increased over the last few years.^34^

### Global and regional mortality due to alcohol-associated cirrhosis

**FIGURE 3:**
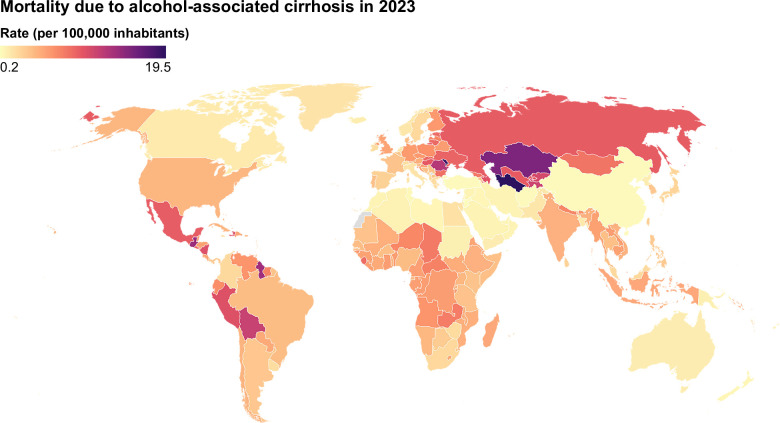
Age-standardized mortality due to alcohol-associated cirrhosis (2023). Data were obtained from the Global Burden of Disease database (https://vizhub.healthdata.org/gbd-results/).

In 2023, a total of 308,801 deaths were estimated due to alcohol-associated cirrhosis globally.^29^^,^^30^ In the same year, the age-standardized mortality due to alcohol-associated cirrhosis was estimated at 3.39 per 100,000 inhabitants (Figure 3). Between 1990 and 2023, the total percentage change in age-standardized mortality due to alcohol-associated cirrhosis decreased by 33%. The differences in age-standardized mortality by sex were even more evident in 2023, with rates estimated at 5.5 and 1.5 per 100,000 inhabitants in men and women, respectively. Between 1990 and 2023, mortality has decreased 32% and 38% in men and women, respectively.^29^^,^^30^

In parallel with alcohol consumption levels, age-standardized mortality due to alcohol-associated cirrhosis was highest in the European WHO Region (5.8 per 100,000) in 2023, followed by the Region of the Americas (5.0 per 100,000 inhabitants) (Figure 3). Conversely, the lowest age-standardized mortality was observed in the Eastern Mediterranean and Western Pacific WHO Regions, with 1.1 and 1.4 per 100,000 inhabitants, respectively. Notably, age-standardized mortality was highest in low and low-middle SDI countries (4.5 and 4.0 per 100,000 inhabitants, respectively), while it was lowest (2.9 and 3.2 per 100,000 inhabitants) in countries with high-middle and high SDI. These findings align with the alcohol harm paradox, where disadvantaged individuals experience higher ALD-related mortality than those with higher socioeconomic status, even with lower levels of alcohol use and a lower prevalence of ALD.^35^

### HCC in ALD and other extrahepatic neoplasms

In 2023, the estimated global incidence of alcohol-associated HCC was 1.2 per 100,000 inhabitants, with a corresponding global age-standardized mortality rate of 1.0 per 100,000 inhabitants.^29^^,^^30^ GLOBOCAN projection data estimate that HCC will account for ~1.37 million deaths worldwide by 2050.^36^ Apart from MASLD, alcohol-associated HCC is the only etiology that has shown a sustained rise in incidence over recent decades, both in the overall population and in early-onset HCC.^37^^,^^38^ Across the WHO regions, the age-standardized incidence of HCC was highest in the African and European Regions (2.1 and 1.7 per 100,000 inhabitants, respectively) and lowest in the Eastern Mediterranean and Western Pacific Regions (0.8 per 100,000 inhabitants for both). The age-standardized incidence of HCC was higher in countries with a low and high SDI (1.4 and 1.3 per 100,000 inhabitants) and lower in those with high-middle and low-middle SDI (0.8 per 100,000 inhabitants for both). Similar patterns were observed for age-standardized mortality due to alcohol-associated HCC, which was higher in low and high SDI countries (1.4 and 1.2 per 100,000 inhabitants) and lower in high-middle and low-middle SDI countries (0.8 per 100,000 inhabitants for both).^29^^,^^30^

The contribution of alcohol to extrahepatic cancers is also important. The International Agency for Research on Cancer (IARC) has classified alcohol as carcinogenic to humans (group 1).^39^ Besides liver cancer, the IARC has identified 6 other solid cancers caused by consumption of alcohol, including oral cavity cancer, pharyngeal cancer, laryngeal cancer, esophageal cancer, colorectal cancer, and breast cancer.^40^ In 2020, about 4.4% of all new cancer cases worldwide were attributable to alcohol, mostly aerodigestive and breast cancers.^2^^,^^10^^,^^41^ Alcohol consumption is substantially linked with cancer burden, and current evidence suggests that there is no threshold of alcohol consumption below which cancer risk does not increase.^42^^,^^43^

### Projections of the mortality due to alcohol-associated cirrhosis to 2050

Using the GBD Foresight visualization data,^44^ we have projected that from 2021 to 2050, the crude mortality of ALD is projected to increase by 76% from 354,200 to 624,150, with significant sex, regional, and sociodemographic variation (Table 1). Regarding sex differences, men will continue to exhibit higher ALD mortality; however, the gender gap is projected to narrow between 2021 and 2050.^11^^,^^45^^–^^47^ By 2050, age-standardized mortality is expected to be 10.3 (95% UI: 8.3–12.6) per 100,000 inhabitants in men and 3.0 (95% UI: 2.2–4.1) in women. Although men bear a greater absolute burden, the annual percentage change (APC) from 2021 to 2050 will be higher in women—1.43% (95% CI: 1.42–1.44)—than in men—1.36% (95% CI: 1.35–1.36)—(both *p*<0.001), reflecting a comparable rate of increase in women (Table 1). The absolute number of deaths is projected to rise more sharply among women (80%) than men (75%) (Table 1). Women exhibit greater biological vulnerability to alcohol-associated harm, which results in a higher risk of major adverse liver outcomes compared with men.^48^^,^^49^

**TABLE 1:** Death, age-standardized death rate, and annual percentage change from 2021 to 2050 of alcohol-associated cirrhosis, by sex, region, and sociodemographic index

	2021 Deaths	2021 ASDR per 100,000 (95% UI)	2050 Deaths	2050 ASDR per 100,000 (95% UI)	2021 to 2050 APC (95% CI)	*p*
Overall	354,200	4.49 (3.76–5.34)	624,150	6.66 (5.31–8.28)	1.37 (1.36–1.38)	<0.001
By sex						
Women	77,790	1.98 (1.57–2.45)	139,780	2.99 (2.16–4.14)	1.43 (1.42–1.44)	<0.001
Men	276,410	6.98 (5.88–8.28)	484,370	10.32 (8.27–12.64)	1.36 (1.35–1.36)	<0.001
By region						
Africa	30,350	2.63 (1.88–3.47)	80,640	3.80 (2.67–4.95)	1.28 (1.26–1.30)	<0.001
Eastern Mediterranean	6680	0.89 (0.63–1.2)	15,170	1.43 (0.95–2)	1.67 (1.66–1.68)	<0.001
Europe	84,030	9 (7.77–10.21)	89,720	9.69 (7.95–11.56)	0.26 (0.25–0.27)	<0.001
The Americas	69,010	6.72 (5.81–7.63)	108,820	9.52 (7.96– 11.2)	1.21 (1.21–1.22)	<0.001
Southeast Asia	124,320	6.02 (4.45–7.74)	249,160	10.91 (7.56–14.74)	2.07 (2.07–2.08)	<0.001
Western Pacific	37,770	1.96 (1.6–2.44)	78,460	4.34 (3.33–5.54)	2.78 (2.76–2.79)	<0.001
By SDI						
Low SDI	32,300	2.89 (2.13–3.76)	67,000	4.12 (2.89–5.48)	1.19 (1.14–1.24)	<0.001
Low-middle SDI	90,020	4.69 (3.4–5.95)	198,730	6.93 (4.88–9.12)	1.39 (1.29–1.49)	<0.001
Middle SDI	103,850	4.24 (3.62–4.98)	214,840	8.37 (6.73–10.31)	2.35 (2.34–2.36)	<0.001
High-middle SDI	66,090	5.07 (4.42–5.76)	58,310	4.87 (3.98–5.77)	0.07 (0.00–0.13)	0.056
High SDI	61,500	5.62 (4.86–6.38)	84,880	7.67 (6.48–9.05)	0.88 (0.86–0.91)	<0.001

*Note:* Data were extracted from https://vizhub.healthdata.org/gbd-foresight/, and the change of trend is assessed by the Jointpoint regression program.

Abbreviations: APC, annual percent change; ASDR, age-standardized death rate; CI, confidence interval; SDI, sociodemographic index; UI, uncertainty interval.

Marked regional differences in ALD mortality trends are projected to be observed. The greatest relative increase will be seen in the Western Pacific Region, with an APC of 2.78% (95% CI: 2.76–2.79; *p*<0.001), where age-standardized mortality would rise from 2.0 (95% UI: 1.6–2.4) in 2021 to 4.3 (95% UI: 3.3–5.5) per 100,000 inhabitants in 2050. Similarly, Southeast Asia is projected to experience a sharp rise, from 6.0 to 10.9 per 100,000, with an APC of 2.07% (95% CI: 2.07–2.08; *p*<0.001) (Table 1). The European Region had the highest age-standardized mortality in 2021 [9.0 (95% UI: 7.8–10.2) per 100,000 inhabitants] and is projected to maintain a high burden in 2050 (9.7; 95% UI: 8.0–11.6), with a more modest APC of 0.26% (95% CI: 0.25–0.27; *p*<0.001). The Region of the Americas and Africa would also face rising trends with APCs of 1.21% and 1.28%, respectively (both *p*<0.001), while the Eastern Mediterranean will have the second-highest APC at 1.67% (95% CI: 1.66–1.68; *p*<0.001) (Table 1). These disparities may be attributed to weaker alcohol control measures and, in some cases, government-endorsed pro-alcohol initiatives—such as reduced taxation on spirits or promotional campaigns aimed at boosting economic revenue through increased alcohol sales—which have contributed to the increasing consumption in certain countries within the Asia-Pacific region.^50^^–^^52^

Middle-SDI countries are projected to experience the steepest rise in ALD mortality, with age-standardized mortality doubling from 4.2 (95% UI: 3.6–5.0) in 2021 to 8.4 (95% UI: 6.7–10.3) in 2050, corresponding to an APC of 2.35% (95% CI: 2.34–2.36; *p*<0.001). Low SDI countries are projected to experience an increase in age-standardized mortality, from 2.9 to 4.1 per 100,000. In contrast, age-standardized mortality in high-middle SDI countries is likely to plateau (APC: 0.07%; 95% CI: 0.00–0.13; *p*=0.056), while high SDI countries will see a modest increase (APC: 0.88%; 95% CI: 0.86–0.91; *p*<0.001) (Table 1). In low and low-middle SDI countries, ALD mortality is projected to increase steadily, driven by gradual increases in alcohol availability as economies develop.^53^ Middle and high-middle SDI countries show contrasting trajectories: middle-SDI regions are projected to experience the sharpest increases in ALD mortality, reflecting increasing alcohol consumption and poor public health implementation. In contrast, high-middle SDI countries appear to be plateauing, likely due to stronger alcohol control policies and maturing health systems that are beginning to mitigate ALD risk.^54^^,^^55^ Together, these patterns highlight both how the burden of alcohol harms changes with changing SDI, and the crucial role of public health policy responses in altering the course of ALD mortality.^56^^,^^57^

## HEALTHCARE COSTS AND HEALTHCARE UTILIZATION

Beyond health impacts, social harms, and crime, alcohol imposes an enormous economic cost on societies. A systematic review of societal cost estimated that alcohol consumption costs countries 1.5%–2.6% of their gross domestic product in economic losses.^58^ Most of these costs are attributable to productivity losses, including workforce absenteeism, unemployment, and reduced output due to alcohol-associated disability and premature mortality.^58^ Moreover, the per-capita economic cost has been estimated at about International Dollar 1300 per adult per year when averaging across countries.^58^ In lower-income settings, these costs are disproportionately burdensome economically, as resources diverted to managing alcohol-associated harms undermine development.^59^ It is important to note that the cost per liter of alcohol consumed, in terms of health burden, is higher in low and middle-income countries than in high-income countries.^1^ This indicates that for an equivalent volume of alcohol, poorer populations suffer greater harm, likely due to weaker health systems, comorbidities, and social determinants that amplify alcohol’s impact.^60^

It is expected that the economic impact of ALD will be substantial and will continue to increase. In the United States, one simulated scenario analysis projected that ALD could generate ~$880 billion in total costs between 2022 and 2040 (including $355 billion in direct medical spending), with annual ALD expenditures rising from ~$31 billion in 2022 to $66 billion by 2040.^61^ A similar pattern is seen in broader alcohol-associated inpatient spending: a recent study estimated that alcohol-associated hospitalizations cost $32.6 billion in 2022 in the United States, indicating increasing costs even after accounting for inflation.^62^

ALD places a substantial burden on patient care and is linked to poor clinical outcomes. In the United States, alcohol-associated hospitalization rates have remained high (around 700 per 100,000 adults per year), while admissions for alcohol-induced medical complications, including alcohol-associated hepatitis and alcohol-associated cirrhosis, increased from about 70–83 per 100,000 between 2016 and 2022.^62^ Over the same interval, outcomes for alcohol-associated admissions worsened, with mean length of stay increasing (~5.6–6.2 d) and in-hospital mortality rising from 2.4% to 3.1%.^62^ Compared with other inpatients, those hospitalized with ALD often require more resources: one comparative analysis found median costs nearly twice those of age-matched and sex-matched non-ALD admissions and longer median stays.^63^ ALD admissions have also been associated with greater acute care needs and much higher mortality, including more frequent transfusion requirements and markedly higher in-hospital death rates (13.2% vs. 0.2% in one series).^63^ Similar patterns have been described in other regions; in Europe, ALD constitutes a dominant share of liver care. In Portugal, alcohol-associated cirrhosis represented 38.6% of liver disease discharges and roughly 43% of inpatient liver-disease costs in the 2000s.^64^

The COVID-19 pandemic appeared to further intensify ALD-related healthcare utilization and adverse outcomes.^20^^,^^65^ Increases in high-risk drinking during the pandemic coincided with rises in severe ALD presentations, advanced fibrosis, and hospital admissions.^20^^,^^24^^,^^66^ In the first pandemic year (2019–2020), ALD mortality in the United States increased by more than 20%, with the greatest relative increases among adults younger than 45 years.^67^ Moreover, sex-stratified analyses showed that during the COVID-19 pandemic period, hospital admission rates for ALD increased more in women than in men.^68^^–^^70^

## SCREENING FOR ALCOHOL INTAKE, AUD, AND ALD

### Quantification of alcohol use and screening for AUD

Routine alcohol use screening is a cornerstone of ALD prevention and supports clinical and epidemiological research.^71^ As noted above, alcohol intake is the main modifiable risk factor for ALD, and—together with liver fibrosis—is also the leading risk factor for liver-related mortality in ALD.^72^^–^^74^ For routine clinical practice, it is important to document current alcohol intake (ideally in grams or standard drinks/week), drinking patterns, and prior alcohol exposure. Given that most patients experience dynamic changes in alcohol use over time, a single, static assessment of alcohol use can misclassify the ALD diagnosis.^13^^,^^75^ In the following paragraphs, we will discuss the principal methods to assess alcohol use and AUD, and the potential implications for epidemiologic estimates. Table 2 summarizes commonly used questionnaires and biomarkers, their detection windows, and test characteristics.

**TABLE 2:** Questionnaires and alcohol biomarkers to identify alcohol exposure or alcohol use disorder in adults

Test/tool	Source/description	Detection window	Cutoff value	Sensitivity	Specificity
Questionnaires					
AUDIT (10-item)^72^	10 questions covering consumption, dependence, and alcohol-associated problems	Past year	>8 (hazardous); >20 (dependence)	38%–73%	89%–97%
AUDIT-C (3-item)^72^	First 3 AUDIT questions on frequency, typical amount, and heavy drinking	Past year	≥4 (men); ≥3 (women)	82%–100% (men); 7%3–97% (women)	28%–91%
NIAAA Single Alcohol Screening Question (SASQ)^72^	“How many times in the past year have you had 5+ (men) or 4+ (women) drinks in a day?“	Past year	≥1 time	73%–88%	74%–100%
Alcohol biomarkers					
Ethyl glucuronide (EtG)^76^^,^^77^	Urine	1–3 d	100–200 ng/mL	89%	99%
	Hair	Months	30 pg/mg	96%	99%
Ethyl sulfate (EtS)^78^^,^^79^	Urine	1–3 d	75 ng/mL	82%	86%
Phosphatidylethanol (PEth)^80^^,^^81^	Blood	2–4 wk	20 ng/mL	73%–100%	90%–96%
Carbohydrate-deficient transferrin (CDT)^82^	Blood	2–3 wk	1.7–2.6%	21%–50%	50%–100%

*Notes:* Because test characteristics vary by clinical setting, reference standard, and cutoff, the sensitivity and specificity values should be interpreted as approximate ranges rather than fixed properties. All alcohol biomarkers need to be interpreted in conjunction with self-reported alcohol intake, and they do not replace a proper interview to quantify alcohol intake in routine clinical practice.

Abbreviations: AUDIT, Alcohol Use Disorders Identification Test; AUDIT-C, Alcohol Use Disorders Identification Test—Consumption; CDT, carbohydrate-deficient transferrin; EtG, ethyl glucuronide; EtS, ethyl sulfate; NIAAA, National Institute on Alcohol Abuse and Alcoholism; PEth, phosphatidylethanol; SASQ, Single Alcohol Screening Question.

Brief validated questionnaires remain the core entry point given their speed, feasibility for universal deployment, cost, and acceptable diagnostic performance in primary care. For instance, the Alcohol Use Disorder Identification Test (AUDIT) is a 10-item screening tool for AUD, while its short version, AUDIT–Consumption (AUDIT-C), includes the first 3 AUDIT items and identifies hazardous drinking.^75^ The National Institute on Alcohol Abuse and Alcoholism (NIAAA) Single Alcohol Screening Question is another useful instrument, in which a positive answer to “How many times in the past year have you had (4 for women or 5 for men) or more drinks in a day?” may trigger further assessment for AUD.^83^ Other instruments, including the Timeline Followback and Lifetime Drinking History (LDH) questionnaires, can provide a more comprehensive quantification of current and past drinking, respectively.^13^ A scoping review of 9 studies (n=6010 participants) found that LDH demonstrates moderate to strong test–retest reliability and concurrent/construct validity.^84^ However, these are not routinely used due to time constraints. There is substantial heterogeneity in the assessment of alcohol use among epidemiological studies.^85^^–^^87^ This heterogeneity arises from differences in measurement tools, definitions, recall periods, and categorization of drinking patterns, jeopardizing adequate comparison between cohorts and regions.

### Alcohol biomarkers

Direct alcohol biomarkers are best viewed as complementary to structured self-report in identifying alcohol exposure.^80^ The major advantage of biomarkers is that they mitigate the well-documented limitations of self-report, particularly in specialty care and transplant contexts. Phosphatidylethanol (PEth) is a direct blood biomarker formed only in the presence of ethanol and is typically detectable over ~2–4 weeks, making it suitable for identifying under-reported use, monitoring abstinence or reduction, and supporting longitudinal care plans in chronic liver disease (Table 2).^13^^,^^80^^,^^88^ In a well-characterized cohort of adults at risk of steatotic liver disease, the use of PEth can increase the diagnosis of ALD 3-fold.^89^ Moreover, PEth has also demonstrated being a better predictor of liver-related events over time than self-reported alcohol intake.^90^

By contrast, ethyl glucuronide (EtG) and ethyl sulfate (EtS) are short-horizon direct metabolites measured most commonly in urine, useful for detecting recent alcohol exposure between 1 and 3 days (Table 2).^28^ Their main limitations are interpretive: EtG in particular can be vulnerable to false positives or negatives under specific circumstances (eg, bacterial infection), and incidental ethanol exposure can matter at very low cutoffs; measuring EtG and EtS together increases confidence.^91^^,^^92^ Finally, carbohydrate-deficient transferrin (CDT) is an established serum biomarker associated with sustained heavy drinking; it can support detection of ongoing heavy consumption but is less sensitive for intermittent heavy episodic drinking and is typically best used in combination with clinical assessment and/or other biomarkers rather than as a stand-alone screen (Table 2).^75^ Of note, a positive alcohol biomarker must always be interpreted in the clinical setting.

Finally, comparing drinking patterns across clinical settings helps define where each screening modality adds the most value. In primary care, unhealthy alcohol use is common but heterogeneous, as many patients with hazardous or episodic patterns of consumption who may not perceive themselves as “problem drinkers,” making brief tools like AUDIT-C well-suited for universal case-finding.^93^ In emergency departments and trauma settings, the prevalence of recent heavy episodic drinking is higher, favoring a combined questionnaire plus blood alcohol concentration (BAC), allowing immediate confirmation to influence care planning.^94^ In liver clinics and transplant programs, sustained heavy drinking, relapse detection, and longitudinal monitoring are central, so PEth is often the most informative single biomarker.^75^ At a population level, heavy episodic drinking remains common in many regions (with marked sex differences in many settings), reinforcing that screening strategies must capture both volume and pattern of consumption, and that effective implementation depends on matching the tool to the clinical question, the time horizon of interest, and the local prevalence of unhealthy use.^95^

The use of alcohol biomarkers, particularly those with a long detection window, demonstrates that individuals often underreport alcohol use across different settings and regions,^96^ with underreporting estimated to occur in up to 55% of patients in routine practice and up to 10% of clinical trials.^97^ This underreporting impacts the accurate diagnosis of ALD and metabolic dysfunction and alcohol-associated liver disease (MetALD). Moreover, alcohol consumption often changes over time, and ~60% of individuals with alcohol consumption within the MetALD and ALD ranges may change their diagnosis category, mostly because of decreasing alcohol use.^98^ Another phenomenon of relevance is the Hawthorne effect, where people change their behavior, often reducing alcohol intake, simply because they know they are being observed or studied. Finally, the assessment of liver fibrosis can also impact alcohol consumption over time.^99^ All the factors highlight the limitations in the identification of individuals with ALD, usually resulting in underestimating the true impact of alcohol intake on liver disease.

## BEST-BUY POLICIES TO REDUCE ALCOHOL INTAKE AND ALD

Alcohol-associated public health policy is a core upstream lever for preventing ALD, because alcohol use is the primary attributable and modifiable driver of ALD at the population level.^100^^–^^102^ In 2018, the WHO consolidated the strongest evidence into the 5-dimensional SAFER framework:^39^^,^^103^ *S*trengthen restrictions on alcohol availability; *A*dvance and enforce drink-driving countermeasures; *F*acilitate access to screening, brief interventions, and treatment; *E*nforce bans or comprehensive restrictions on advertising, sponsorship, and promotion; and *R*aise prices through excise taxes and pricing policies.^39^^,^^103^ WHO subsequently aligned this approach with the Global Alcohol Action Plan 2022–2030, endorsed by Member States, which aims to reduce per-capita consumption by 20% by 2030 through the scaled implementation of high-impact interventions.^104^

Among SAFER components, pricing measures have the most robust and reproducible benefits because they reduce the affordability of alcoholic beverages (Table 3).^115^ For instance, Scotland enacted minimum unit pricing (MUP) in 2018, which has been associated with a 13.4% reduction in alcohol-specific deaths and a 4.1% reduction in hospitalizations, alongside decreases in ALD-related deaths (11.7%) and ALD-related hospitalizations (9.8%).^105^ Similar natural-experiment evidence from British Columbia shows that raising minimum alcohol prices is associated with rapid and sustained decreases in alcohol-attributable admissions,^116^ whereas Finland’s large alcohol tax reduction illustrates the reverse: increased affordability was followed by marked increases in alcohol-associated harm, including liver mortality, supporting a causal affordability–harm relationship.^107^ At the macro level, cross-jurisdictional analyses further support this policy gradient: stronger composite alcohol-policy environments across countries and stronger state-level policy implementation within the United States are each associated with lower alcohol-associated harms and alcohol-associated cirrhosis mortality.^108^

**TABLE 3:** World Health Organization SAFER “Best-Buy” alcohol policies and their impact on alcohol-associated harms and alcohol-associated liver disease outcomes

SAFER component	Policy intervention	Location tested	Effect on alcohol harms and/or liver outcomes
Raise prices	Minimum unit pricing (MUP)^105^	Scotland	Post-implementation evaluations reported reductions in alcohol-specific deaths and alcohol-attributable hospitalizations, with parallel improvements in liver-related endpoints in controlled time-series analyses.
Raise prices	Minimum alcohol price increases^106^	Canada (British Columbia)	Natural-experiment analyses associated higher minimum prices with rapid and sustained decreases in acute and chronic alcohol-attributable hospital admissions.
Raise prices	Large excise tax reduction (reverse natural experiment)^107^	Finland	A major alcohol tax cut increased affordability and was followed by increased alcohol-associated harm, including higher liver mortality, supporting an affordability–harm relationship.
Raise prices/policy strength	Alcohol policy strength (state-level composite/implementation index)^108^	United States (state-level)	Stronger state alcohol-policy environments were associated with lower alcohol-associated harms, including lower alcohol-associated cirrhosis mortality, supporting a policy-strength gradient.
Strengthen availability	Outlet density/physical availability^108^	United States (ecologic/policy analyses)	Greater physical availability (including higher outlet density) was associated with worse liver outcomes over time, supporting licensing and sales-hour restrictions as preventive levers.
Raise prices + strengthen availability + enforce marketing restrictions (policy package)	Comprehensive reform package (tax increases + reduced availability + marketing restrictions)^109^	Lithuania	National reforms (2016–2018) were associated with steep declines in per-capita consumption and reductions in alcohol-associated mortality, illustrating additive benefits of multi-component SAFER implementation.
Raise prices + strengthen availability (policy package)	Multi-component alcohol-control measures (taxation/enforcement/availability of cheap alcohol)^110^	Russia	Policy strengthening aligned with substantial declines in alcohol-attributable mortality, including liver deaths; policy weakening periods coincided with worse outcomes, supporting sustained portfolios.
Enforce marketing restrictions	Advertising restrictions/bans^111^	Multiple countries	Econometric cross-national work suggests advertising restrictions are associated with modest reductions in per-capita consumption, though effect sizes vary and co-interventions can limit attribution.
Raise prices (evidence synthesis)	Minimum pricing (systematic review)^112^	Multiple countries	Evidence syntheses generally find that minimum pricing reduces consumption and alcohol-attributable harms across settings, with magnitude varying by design and endpoints.
Raise prices (modeling evidence)	Minimum pricing (modeling)^113^	South Africa	Modeling studies project that minimum pricing can reduce consumption and avert alcohol-attributable deaths over time, particularly affecting heavy drinking patterns.
Cross-cutting implementation	Industry interference delaying policy implementation (eg, warning labels)^114^	Ireland	Implementation delays and dilution of evidence-based policy due to industry pressure highlight that real-world impact depends on governance, accountability, and protection from conflicts of interest.

Abbreviation: SAFER, Strengthen restrictions on alcohol availability; Advance and enforce drink-driving countermeasures; Facilitate access to screening, brief interventions, and treatment; Enforce bans or comprehensive restrictions on advertising, sponsorship, and promotion; Raise prices through excise taxes and pricing policies.

Beyond price, availability restrictions represent another high-impact lever (Table 3). Studies linking alcohol outlet density to cirrhosis mortality confirm that greater physical availability is associated with worse liver outcomes over time, supporting restrictions via licensing and sales-hour controls.^108^ Scaling screening, brief interventions, and referral to treatment can also reduce consumption when implemented at sufficient coverage in primary care, but their impact depends on workforce training, workflow integration, and treatment capacity.^117^

Importantly, evidence from countries implementing policy packages suggests additive benefits when multiple SAFER components are combined. Russia’s multi-component alcohol-control measures resulted in substantial declines in alcohol-attributable mortality, including liver deaths, while periods of weakened policy coincided with worsening outcomes, underscoring the value of a sustained, coordinated policy strategy.^110^ Similarly, Lithuania’s 2016–2018 reform package combining tax increases, reduced availability, and marketing restrictions was associated with steep declines in consumption and alcohol-associated mortality.^109^ These findings align with an ecological study in Latin America that evidenced a significant association between a national plan on alcohol and the prevalence of ALD.^57^

Policies targeting alcohol marketing are also directionally supportive. Cross-national econometric work suggests that comprehensive advertising restrictions are associated with modest reductions in per-capita consumption, although effect sizes vary and attribution can be limited by co-interventions.^111^ Evidence syntheses further support minimum pricing as a consistent population tool across settings, with reductions in consumption and alcohol-associated harms observed in natural studies and supported by modeling studies where real-world data are limited.^112^^,^^118^ Finally, because policy effectiveness is often constrained more by implementation than evidence, industry interference that delays or dilutes regulation, illustrated by postponed alcohol warning-label implementation in Ireland, highlights the need for transparent governance, protection from conflicts of interest, and accountable delivery to achieve equitable and sustained reductions in ALD burden.^114^

The tragedy of alcohol-associated harms and deaths is that there are clear evidence-based policy solutions that are well known but poorly implemented in most jurisdictions. Alcohol policy progress is often slowed, weakened, or blocked by alcohol industry interference, including lobbying to delay or dilute evidence-based regulations, as illustrated by Ireland’s experience with health warning labeling, in which implementation of prominent health warning labels legislated under the Public Health (Alcohol) Act (2018) was postponed following sustained industry pressure and trade-related threats. Taken together, this evidence supports a pragmatic conclusion for ALD prevention: jurisdictions achieve the largest and most equitable reductions in alcohol harm when they implement SAFER as a coherent package—anchored by price and availability levers, reinforced by marketing controls and drink-driving enforcement, and coupled with accessible screening and treatment—while insulating politicians and policymaking from the influence of the alcohol industry, and ensuring sustained, accountable delivery at scale.

## LOCAL AND INNOVATIVE INITIATIVES TO IDENTIFY INDIVIDUALS AT RISK OF ALD

**FIGURE 4:**
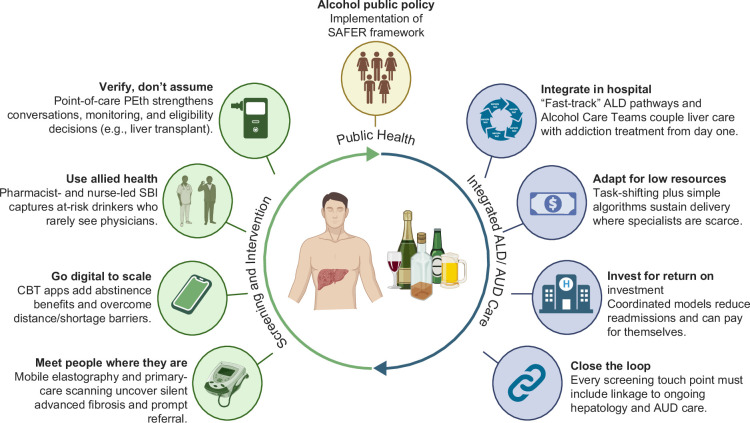
Local and innovative initiatives to identify and manage ALD risk. The figure highlights community case-finding (mobile/primary care transient elastography), scalable digital CBT for AUD, allied-health screening and brief intervention, hospital fast-track ALD pathways/Alcohol Care Teams, and low-resource adaptations including task-shifting and point-of-care PEth to support linkage and integrated ALD–AUD care. Abbreviations: ALD, alcohol-associated liver disease; AUD, alcohol use disorder; CBT, cognitive behavioral therapy; PEth, phosphatidylethanol; SAFER, Strengthen restrictions on alcohol availability; Advance and enforce drink-driving countermeasures; Facilitate access to screening, brief interventions, and treatment; Enforce bans or comprehensive restrictions on advertising, sponsorship, and promotion; Raise prices through excise taxes and pricing policies; SBI, screening and brief intervention.

To complement population-level policies, granular, locally-led innovations are improving how ALD is identified and managed across settings (Figure 4). Community-based screening vans that bring transient elastography to town centers and workplaces have reached people who rarely seek care; the South West England “Alright My Liver?” pilot reported high acceptability and detection of previously unrecognized advanced fibrosis.^119^ Beyond vans, primary care programs that embed transient elastography have demonstrated feasibility and longer-term utility, reinforcing the value of community case-finding.^120^ Such outreach can facilitate earlier diagnosis and linkage to hepatology and addiction services.^119^

In terms of treatment of AUD, digital cognitive-behavioral-therapy (CBT) programs can expand healthcare access. In a U.S. randomized trial, adding a web-based CBT program (CBT4CBT) to usual care significantly increased abstinent days at 6 months compared with standard care.^121^ A 12-week mobile CBT app from South Korea similarly improved end-of-treatment abstinence and reduced craving compared with face-to-face counseling.^122^ Allied-health delivery models extend reach: in Australian community pharmacies, brief training enabled pharmacists to screen with AUDIT and provide brief advice with high acceptability—an opportunity to intervene early among individuals who may not present to physicians.^123^

Nurse-led and pharmacist-led screening and brief intervention in primary care offers a pragmatic approach to attenuating progression to ALD when coupled with clear referral and follow-up.^123^ Within hospitals, “fast-track” ALD pathways link hepatology and addiction care early during admission and through discharge. A U.S. multidisciplinary clinic that enrolled inpatients with alcohol-associated hepatitis or cirrhosis into integrated hepatology–addiction follow-up reported improved liver function and markedly fewer emergency visits/readmissions at 6 months.^124^ In the United Kingdom, hospital-based Alcohol Care Teams operationalize similar principles at scale and are associated with reductions in admissions and mortality related to alcohol.^125^

In low-resource settings, task-shifting and point-of-care diagnostics adapt these innovations (Figure 4). In Mozambique, primary-care nurses and psychiatric technicians delivered tablet-supported brief motivational interventions with good fidelity, demonstrating the feasibility of decentralized care.^126^ PEth, a direct alcohol biomarker measurable from blood samples, provides a broad detection window to verify abstinence and support decision-making.^127^ Early primary-care experience indicates PEth availability increases clinicians’ confidence and prompts alcohol discussions.^128^ Collectively, these initiatives—mobile liver assessments, digital therapeutics, allied-health SBIs, hospital fast-track pathways, and low-resource adaptations—offer practical, scalable levers to strengthen ALD care, align with integrated ALD–AUD management frameworks, and improve outcomes.^129^

## FUTURE DIRECTIONS AND KNOWLEDGE GAPS

Several priorities must be addressed to improve the global evidence base and clinical impact of ALD epidemiology. First, harmonized ALD registries are needed across regions and health-system levels, using standardized case definitions, fibrosis staging frameworks, and longitudinal outcome ascertainment.^20^^,^^130^ Registries should capture alcohol exposure with explicit specification of time horizon (recent vs. sustained), pattern (heavy episodic vs. daily heavy use), and treatment engagement, while integrating core liver endpoints (decompensation, HCC, transplant, mortality) and extrahepatic outcomes that shape prognosis (cardiovascular disease, infections, malignancy).^27^^,^^131^^,^^132^ Interoperability with electronic health records and linkage to vital statistics would enable near–real-time monitoring and reduce reliance on cross-sectional estimates.

Second, enhanced surveillance in low- and middle-income countries remains a critical gap, given that they showed a disproportionate growing burden of ALD.^34^^,^^133^ Many countries face limited access to diagnostic tools (elastography, patented serum panels), constrained laboratory capacity, and incomplete cause-of-death coding, resulting in systematic underestimation of ALD burden.^29^^,^^134^^,^^135^ Pragmatic and adapted surveillance should prioritize scalable elements: structured alcohol-use assessment, routine laboratories enabling FIB-4 calculation, sentinel-site elastography, and linkage to hepatitis and metabolic disease programs.^136^^–^^138^ Implementation must be paired with workforce development (task shifting, training) and sustainable financing models for diagnostics and referral pathways.^71^^,^^139^^,^^140^

Third, the field requires rigorous evaluation of integrated genetic and metabolomic risk scores to enable precision prevention. Polygenic risk scores and metabolomic signatures could help identify individuals at disproportionate risk for advanced fibrosis at a given exposure level, but major questions remain around transferability across ancestries, calibration in admixed populations, ethical deployment, and whether risk disclosure improves outcomes without exacerbating stigma.^141^^–^^143^

Finally, PEth-based screening needs robust implementation research. Key gaps include optimal cutoffs by clinical context, sampling frequency for monitoring, handling discordant self-report and biomarker results, patient-centered communication strategies, and comparative effectiveness studies testing whether PEth-guided pathways improve abstinence, reduce decompensation, and enhance equity, especially when embedded within integrated addiction–hepatology care models.^144^^,^^145^

Finally, the most effective and least costly measures to reduce alcohol harms, including the entire spectrum of ALD and death, are preventative, based on the SAFER measures proposed by WHO. Implementation of preventative measures should be a key target of clinical societies and organizations.

## CONCLUSIONS

ALD is a major and increasing cause of preventable liver-related morbidity and mortality worldwide, amplified by diagnostic under-detection and unequal access to effective treatment and surveillance. Clinicians should adopt risk-stratified pathways that combine validated alcohol-use screening with staged fibrosis assessment and ensure rapid linkage to evidence-based alcohol treatment and liver care. Policymakers must implement high-impact alcohol control measures while financing scalable detection and integrated care to reduce the global ALD burden. Researchers should prioritize harmonized registries, surveillance platforms, and trials of biomarker-guided and precision-risk strategies that measure meaningful clinical outcomes and equity.
